# Supplementary material for: Characterizing large-scale quantum computers via cycle benchmarking
Source: Nat Commun. 2019 Nov 25;10:5347. doi: 10.1038/s41467-019-13068-7 (PMC6877623; doi:10.1038/s41467-019-13068-7)
Supplement: Supplementary file 1 — Supplementary Information [file 41467_2019_13068_MOESM1_ESM.pdf]

Supplementary Information for: Characterizing large-scale quantum computers via cycle benchmarking

Erhard and Wallman et al.

Supplementary Note 1. Mathematical assumptions

In this section, we specify the state preparation and measurement (SPAM) procedures, obtain expressions for the expected values of steps in the protocol over the set of all Pauli matrices $\mathsf{P}^{\mathsf{N}} = \{\mathsf{I}, \mathsf{X}, \mathsf{Y}, \mathsf{Z}\}^{\otimes \mathsf{N}}$, and analyze the uncertainties in experimental estimates of those expected values. We conclude by giving a simple expression for the ideal MS gate that facilitates the calculation of $\mathcal{C}(P)$.

For this appendix only, we abuse notation slightly by implicitly defining the channel $\mathcal{P}(A) = PAP^\dagger$ for any Pauli matrix P, so that we can use expressions such as $\sum_P \mathcal{P}$.

We begin by specifying the mathematical assumptions we use in our analysis. We assume that initializing N ions into the ground state corresponds to preparing a mixed state ρ that is independent of any subsequent control operations to be applied. We assume that measuring the ions in the computational basis corresponds to performing a fixed positive-operator-valued measurement (POVM) that depends only upon the number of ions in the trap and not on any prior control operations. We assume that the noise in our implementations of a cycle is Markovian on the timescale of the cycle and is independent and identically distributed each time a cycle is applied. These three assumptions are standard in benchmarking and tomography literature.

Finally, we also assume that the noise in a cycle of independent single-qubit gates is independent of the specific single-qubit gates being implemented. Specifically, we assume that the noisy Markovian implementation $\tilde{\mathcal{C}}$ of a cycle $\mathcal{C}$ of single-qubit gates can be written as $\tilde{\mathcal{C}} = \mathcal{A}\mathcal{C}$ for some fixed completely positive and trace-preserving map $\mathcal{A}$. The assumption of gate-independent noise on the random Pauli gates is weaker than the corresponding assumption in randomized benchmarking, namely, that the noisy implementation of *any* N-qubit Clifford gate $\mathcal{C}_N$ can be written as $\tilde{\mathcal{C}}_N = \mathcal{A}\mathcal{C}_N$, independent of the number of entangling gates required to implement $\mathcal{C}_N$. We expect that this assumption can be further relaxed using the analysis of Ref. [1] at the cost of more cumbersome notation. This will be subject of future research.

Supplementary Note 2. State preparation and measurement procedures

In our experiment, we can only directly perform noisy preparations and measurements in the N-qubit computational basis $\{|z\rangle : z \in \mathbb{Z}_2^N\}$. We now specify the basis changes and coarse graining we use to perform other preparations and measurements. For an N-qubit matrix Q (e.g., P, $\mathcal{C}(P)$ from the main text), let $\mathcal{B}_Q$ rotate the computational basis to an eigenbasis of Q such that

$$\sum_{z \in \mathbb{Z}_2^N} \mathrm{Tr}\left[\mathcal{B}_Q(|z\rangle\langle z|)Q\right] \mathcal{B}_Q(|z\rangle\langle z|) = Q. \tag{1}$$

For the processes we investigated, $\mathcal{C}(P)$ is always an N-qubit Pauli matrix. Therefore, we only need to prepare eigenstates of Pauli matrices P and measure the expectation value of Pauli matrices $\mathcal{C}(P)$. Consequently, our SPAM procedures are fully specified by defining $\mathcal{B}_Q$ for arbitrary Pauli matrices Q. We choose to construct the $\mathcal{B}_Q$ out of local Clifford operators to maximize the SPAM coefficients (which results in a smaller statistical uncertainty). Specifically, let $P|_j$ denote the jth tensor factor of a matrix, $\mathcal{A}_I = \mathcal{A}_Z = \mathcal{I}$ and

$$\mathcal{A}_X(Z) = X, \quad \mathcal{A}_X(X) = Y$$
$$\mathcal{A}_Y(Z) = Y, \quad \mathcal{A}_Y(Y) = X.$$

Then we choose the basis-changing gate for an N-qubit Pauli matrix Q to be

$$\mathcal{B}_Q = \bigotimes_{j=1}^{N} \mathcal{A}_{Q|_j}. \tag{2}$$

Note that the basis changing procedure is independent of the sign of Q.

We now specify the coarse-graining procedure we use to measure the expectation value of observables. Suppose a system is in a state ρ and let $\Pr(\mathrm{z}|\mathrm{Q})$ be the probability of observing the computational basis outcome z after applying the process $\mathcal{B}_Q^\dagger$. One measures the expectation value of Q [e.g., $Q = \mathcal{C}(P)$] by applying $\mathcal{B}_Q^\dagger$, measuring in the computational basis, and averaging the probabilities of the outcomes weighted by the coefficients $\mathrm{Tr}\left[\mathcal{B}_Q(|z\rangle\langle z|)Q\right]$, where the weights are computed from the ideal quantities. From Supplementary Equation (1) and by the linearity of the trace,

$$\mathrm{Tr}[Q\rho] = \sum_{z \in \mathbb{Z}_2^N} \mathrm{Tr}\left[\mathcal{B}_Q(|z\rangle\langle z|)Q\right] \Pr(\mathrm{z}|\mathrm{Q}). \tag{3}$$

Note that as we average the relative frequencies over all outcomes and $\mathrm{Tr}\left[\mathcal{B}_Q(|z\rangle\langle z|)Q\right]$ is in the unit disc, the number of measurements required to estimate the expectation value of Q to a fixed additive precision is independent of the number of qubits N by a standard application of, e.g., Hoeffding's inequality [2].

The above estimation procedure will include several sources of SPAM error per qubit, including errors in qubit initialization, measuring qubits in the computational basis, and in the local processes used to change the basis. Consequently, a protocol has to be robust to SPAM errors to provide a practical characterization of a multi-qubit gate.

Supplementary Note 3. Modelling the decay as a function of the sequence length

We now determine the expected value of $\sum_{l=1}^{L} f_{P,m,l}/L$ for fixed values of P and m underthe assumptions specified in Supplementary Note 1.

Theorem 1. *Let $\mathcal{G}$ be a Clifford cycle and $\tilde{\mathcal{G}}$ be an implementation of $\mathcal{G}$ with Markovian noise. Suppose there exists a process $\mathcal{A}$ such that $\tilde{\mathcal{R}} = \mathcal{A}\mathcal{R}$ for any Pauli process $\mathcal{R}$. Then for a fixed Pauli matrix P and positive integer m, the expected value of $f_{P,m,l}$ from step 3c of the protocol over all random Pauli processes $\mathcal{R}_0, \ldots, \mathcal{R}_m$ is*

$$\langle f_{P,m,l}\rangle = \beta \prod_{j=0}^{m-1} F_{\mathcal{G}^j(P)}(\mathcal{E},\mathcal{I}),$$

where $\mathcal{E} = \mathcal{G}^\dagger\tilde{\mathcal{G}}\mathcal{A}$ and β is a scalar that depends only on P and $\mathcal{G}^m(P)$. Moreover, $\beta = 1$ in the absence of SPAM errors.

Proof. Substituting $\tilde{\mathcal{R}}_i = \mathcal{A}\mathcal{R}_i$ into the noisy version of Eq. (5) in the main text (i.e., overset each operator with a $\sim$), the average superoperator applied over all sequences for a fixed choice of random sequences is

$$\tilde{\mathcal{C}} = \mathcal{A}\mathcal{R}_m\tilde{\mathcal{G}}\ldots\mathcal{A}\mathcal{R}_1\tilde{\mathcal{G}}\mathcal{A}\mathcal{R}_0. \tag{4}$$

Inserting $\mathcal{G}\mathcal{G}^\dagger$ between the ideal Pauli processes $\mathcal{R}_i$ and the adjacent $\tilde{\mathcal{G}}$ gives

$$\tilde{\mathcal{C}} = \mathcal{A}\mathcal{R}_m\mathcal{G}\mathcal{E}\ldots\mathcal{R}_1\mathcal{G}\mathcal{E}\mathcal{R}_0 \tag{5}$$

where $\mathcal{E} = \mathcal{G}^\dagger\tilde{\mathcal{G}}\mathcal{A}$. We can now do a standard relabelling of the randomizing gates to obtain a twirl by setting $\mathcal{T}_0 = \mathcal{R}_0$ and recursively defining

$$\mathcal{R}_i = \mathcal{T}_i\mathcal{G}\mathcal{T}_{i-1}^\dagger\mathcal{G}^\dagger \tag{6}$$

for $i > 0$. With this relabelling,

$$\tilde{\mathcal{C}} = \mathcal{A}\mathcal{T}_m\mathcal{G}\mathcal{T}_{m-1}^\dagger\mathcal{E}\mathcal{T}_{m-1}\ldots\mathcal{T}_1^\dagger\mathcal{E}\mathcal{T}_1\mathcal{G}\mathcal{T}_0^\dagger\mathcal{E}\mathcal{T}_0. \tag{7}$$

The $\mathcal{T}_i$ are all Pauli processes because $\mathcal{G}\mathcal{P}\mathcal{G}^\dagger$ is a Pauli process for any Pauli process $\mathcal{P}$ and any Clifford process $\mathcal{G}$. Moreover, the $\mathcal{T}_i$ are uniformly random because the Pauli processes are sampled uniformly at random and form a group. Therefore averaging independently over all $\mathcal{T}_0, \ldots, \mathcal{T}_{m-1}$ for a fixed choice of $\mathcal{T}_m$ results in the effective superoperator

$$\mathcal{A}\mathcal{T}_m(\mathcal{G}\tilde{\mathcal{E}})^m, \tag{8}$$

where

$$\tilde{\mathcal{E}} = 4^{-N}\sum_{P\in\mathsf{P}^{\mathsf{N}}}\mathcal{P}^\dagger\mathcal{E}\mathcal{P}. \tag{9}$$

Now note that $\tilde{\mathcal{E}}$ is invariant under conjugation by Pauli operators and so $\tilde{\mathcal{E}}(Q) \propto Q$ for all $Q \in \mathsf{P}^{\mathsf{N}}$ [3]. As the Pauli matrices form a trace-orthogonal basis for the set of matrices,

$$\begin{aligned}\tilde{\mathcal{E}}(Q) &= 2^{-N}\operatorname{Tr}\left[Q^\dagger\tilde{\mathcal{E}}(Q)\right]Q \\ &= 4^{-N}\sum_{P\in\mathsf{P}^{\mathsf{N}}}2^{-N}\operatorname{Tr}\left[Q\mathcal{P}^\dagger\mathcal{E}\mathcal{P}(Q)\right]Q \\ &= 4^{-N}\sum_{P\in\mathsf{P}^{\mathsf{N}}}2^{-N}\operatorname{Tr}\left[\mathcal{P}(Q)\mathcal{E}\mathcal{P}(Q)\right]Q \\ &= 4^{-N}\sum_{P\in\mathsf{P}^{\mathsf{N}}}2^{-N}\operatorname{Tr}\left[Q\mathcal{E}(Q)\right]Q \\ &= F_Q(\mathcal{E},\mathcal{I})Q,\end{aligned} \tag{10}$$

for any $Q \in \mathsf{P}^{\mathsf{N}}$, where we have used the fact that $\mathcal{P}(Q) = PQP^\dagger = \pm Q$ for any Pauli matrices P, Q and Eq. (2) in the main text .

For any two Pauli matrices $P, Q \in \mathsf{P}^{\mathsf{N}}$, let

$$\eta(Q,P) = \begin{cases}1 & \text{if } QP = PQ \\ -1 & \text{otherwise.}\end{cases} \tag{11}$$

Then, from Supplementary Equation (8) with $P' = \mathcal{G}^m(P)$ for convenience, the expected outcome of the ideal circuit is $\mathcal{C} = \eta(T_m, P)P'$. Now note that under measurement errors and noisy changes of basis [i.e., errors in the $\Pr(z|Q)$] and folding the residual $\mathcal{A}$ into the measurement, Supplementary Equation (3) gives the expectation value of some operator $\tilde{P}'$ (which is not uniquely defined). Since only the weights in Supplementary Equation (3) depend on the sign of P' and are calculated from the ideal expressions, the noisy measurement for $-P'$ gives the expectation value of $-\tilde{P}'$ by linearity.

Let ρ be the prepared state after applying a noisy change of basis. Then the expectation value of $f_{P,m,l}$ in step 3c over all sequences is

$$\begin{aligned}\langle f_{P,m,l}\rangle &= 4^{-N}\sum_{T_m\in\mathsf{P}^{\mathsf{N}}}\eta(T_m,P')\operatorname{Tr}\left[\mathcal{T}_m^\dagger(\tilde{P}')(\mathcal{G}\tilde{\mathcal{E}})^m(\rho)\right] \\ &= \alpha_P\operatorname{Tr}\left[P'(\mathcal{G}\tilde{\mathcal{E}})^m(\rho)\right]\end{aligned} \tag{12}$$

by Lemma 2 below, where $\alpha_P = 2^{-N}\operatorname{Tr}[P\tilde{P}']$ is 1 in the absence of errors.

Expanding $\rho = \sum_{Q\in\mathsf{P}^{\mathsf{N}}}\rho_Q Q$ and noting that $\mathcal{G}$ is a Clifford cycle, Supplementary Equation (12) reduces to

$$\langle f_{P,m,l}\rangle = \sum_{Q\in\mathsf{P}^{\mathsf{N}}}\alpha_P\rho_Q\operatorname{Tr}\left[P'\mathcal{G}^m(Q)\right]\prod_{j=0}^{m-1}F_{\mathcal{G}^j(Q)}(\mathcal{E},\mathcal{I}). \tag{13}$$

As the Pauli matrices are trace-orthogonal and $P' = \mathcal{G}^m(P)$, $\operatorname{Tr}\left[\mathcal{G}^m(Q)P'\right] = 2^N\delta_{Q,P}$. Therefore

$$\langle f_{P,m,l}\rangle = 2^N\alpha_P\rho_P\prod_{j=0}^{m-1}F_{\mathcal{G}^j(P)}(\mathcal{E},\mathcal{I}), \tag{14}$$

where $\rho_P = 2^{-N}$ in the absence of SPAM errors, so that $\beta = 2^N\alpha_P\rho_P = 1$ in the absence of SPAM errors. □

In the above proof, we make use of the following lemma proven and applied to randomized benchmarking in Ref. [4].

Lemma 2. *For any matrix M and any Pauli matrix P,*

$$4^{-N}\sum_{Q\in\mathsf{P}^{\mathsf{N}}}\eta(Q,P)\mathcal{Q}(M)=2^{-N}\,\mathrm{Tr}\,[PM]\,P.$$

Proof. As the Pauli matrices form an orthogonal basis for the space of matrices, we can write

$$M=\sum_{R\in\mathsf{P}^{\otimes\mathsf{N}}}m_R R, \tag{15}$$

where $m_R=2^{-N}\,\mathrm{Tr}(RM)$. As $\mathcal{Q}(R)=\eta(Q,R)R$ for any Pauli matrix R,

$$4^{-N}\sum_{Q\in\mathsf{P}^{\mathsf{N}}}\eta(Q,P)\mathcal{Q}(M)=\sum_{R\in\mathsf{P}^{\otimes\mathsf{N}}}m_R(\eta_P\cdot\eta_R)R \tag{16}$$

by linearity, where

$$\eta_P\cdot\eta_R=4^{-N}\sum_{Q\in\mathsf{P}^{\mathsf{N}}}\eta(Q,R)\eta(P,R). \tag{17}$$

As $\eta(Q,P)$ is a real 1-dimensional representation of the Pauli group for any fixed Pauli matrix P and $\eta(Q,P)$ and $\eta(Q,R)$ are inequivalent as representations for $P\neq R$,

$$4^{-N}\sum_{Q}\eta(P^{(m)},Q)\eta(P,Q)=\delta(P,R) \tag{18}$$

by Schur's orthogonality relations. □

Supplementary Note 4. Estimating the process fidelity

We now prove that the expectation value of Eq. (7) in the main text provides an accurate, yet conservative, estimate of the process fidelity in Eq. (4) in the main text under the same assumptions as in Eq. (2) in the main text .

Theorem 3. *Let*

$$\hat{F}=4^{-N}\sum_{P\in\mathsf{P}^{\mathsf{N}}}\left(\frac{\langle f_{P,m_2,l}\rangle}{\langle f_{P,m_1,l}\rangle}\right)^{\frac{1}{m_2-m_1}}$$

be the expected outcome of the cycle benchmarking protocol over all randomizations. Let $\mathcal{G}$ be a Clifford cycle and $\tilde{\mathcal{G}}$ be an implementation of $\mathcal{G}$ with Markovian noise. Suppose there exists a process $\mathcal{A}$ such that $\tilde{\mathcal{R}}=\mathcal{A}\mathcal{R}$ for any Pauli process $\mathcal{R}$. Then $\hat{F}\leq F_{\mathrm{RC}}(\tilde{\mathcal{G}},\mathcal{G})$ and

$$\hat{F}-F_{\mathrm{RC}}(\tilde{\mathcal{G}},\mathcal{G})=\mathcal{O}\left([1-F_{\mathrm{RC}}(\tilde{\mathcal{G}},\mathcal{G})]^2\right).$$

Proof. First, recall that the process fidelity is linear and for any unitary process $\mathcal{U}$,

$$F(\tilde{\mathcal{G}},\mathcal{U})=F(\mathcal{U}^\dagger\tilde{\mathcal{G}},\mathcal{I}).$$

Therefore from Eq. (4) in the main text ,

$$\begin{aligned}F_{\mathrm{RC}}(\tilde{\mathcal{G}},\mathcal{G})&=4^{-N}\sum_{R\in\mathsf{P}^{\mathsf{N}}}F(\tilde{\mathcal{G}}\tilde{\mathcal{R}},\mathcal{G}\mathcal{R})\\&=4^{-N}\sum_{R\in\mathsf{P}^{\mathsf{N}}}F(\mathcal{R}\mathcal{G}^\dagger\tilde{\mathcal{G}}\mathcal{A}\mathcal{R},\mathcal{I})\\&=F(\tilde{\mathcal{E}},\mathcal{I}).\end{aligned}$$

Moreover, $F(\mathcal{E},\mathcal{I})=F(\tilde{\mathcal{E}},\mathcal{I})$ by Eq. (1) in the main text and Supplementary Equation (10), and so we will prove statements for $F(\mathcal{E},\mathcal{I})$.

Now fix a Pauli matrix P and note that if m_1 and $m_2=m_1+\delta m$ are chosen so that $P'=\mathcal{G}^{m_2}(P)=\mathcal{G}^{m_1}(P)$ (guaranteed by step 2 of the protocol), then

$$\left(\frac{\langle f_{P,m_2,l}\rangle}{\langle f_{P,m_1,l}\rangle}\right)^{1/\delta m}=\prod_{j=0}^{\delta m-1}F_{\mathcal{G}^j(P')}(\mathcal{E},\mathcal{I})^{1/\delta m} \tag{19}$$

by Theorem 1, as the scalar is the same for m_1 and m_2. That is, the terms being averaged over in Eq. (7) in the main text are themselves geometric means of $F_Q(\tilde{\mathcal{E}},\tilde{\mathcal{I}})$ for different Pauli matrices Q obtained by applying $\mathcal{G}$ to the sampled P. Formally, let $w(Q|P',\delta m)$ be the relative frequency of Q in the list $(\mathcal{G}^j(P'):j=0,\ldots,\delta m-1)$. Then

$$\left(\frac{\langle f_{P,m_2,l}\rangle}{\langle f_{P,m_1,l}\rangle}\right)^{1/\delta m}=\prod_{Q\in\mathsf{P}^{\mathsf{N}}}F_Q(\mathcal{E},\mathcal{I})^{w(Q|\mathcal{G}^{m_1}(P),\delta m)} \tag{20}$$

By the inequality of the weighted arithmetic and geometric means,

$$\left(\frac{\langle f_{P,m_2,l}\rangle}{\langle f_{P,m_1,l}\rangle}\right)^{1/\delta m}\leq\sum_{Q\in\mathsf{P}^{\mathsf{N}}}w(Q|P,\delta m)F_Q(\mathcal{E},\mathcal{I}). \tag{21}$$

As $\mathcal{G}$ is a Clifford matrix, $\sum_{P\in\mathsf{P}^{\mathsf{N}}}w(Q|P,\delta m)=1$ for all Pauli matrices Q. Therefore summing Supplementary Equation (21) over all input Pauli matrices P gives $\hat{F}\leq F(\mathcal{E},\mathcal{I})$. To prove the approximate statement, let $r_Q=1-F_Q(\mathcal{E},\mathcal{I})$. Expanding Supplementary Equation (20) to second order in the r_Q gives

$$\left(\frac{\langle f_{P,m_2,l}\rangle}{\langle f_{P,m_1,l}\rangle}\right)^{1/\delta m}=1-\sum_{Q\in\mathsf{P}^{\mathsf{N}}}w(Q|P,\delta m)r_Q+\mathcal{O}(r_Q^2).$$

The approximate claim then holds as $\mathcal{O}(r_Q^2)=\mathcal{O}([1-F(\mathcal{E},\mathcal{I})]^2)$ by Lemma 4 below. □

Lemma 4. *For any completely positive and trace-preserving map $\mathcal{E}$ and any Pauli matrix P,*

$$0\leq 1-F_P(\mathcal{E},\mathcal{I})\leq 2-2F(\mathcal{E},\mathcal{I}).$$

Proof. Note that Supplementary Equation (10) holds for any completely positive and trace preserving map $\mathcal{E}$ with $\tilde{\mathcal{E}}$ as defined in Supplementary Equation (9). In particular, $F_P(\tilde{\mathcal{E}},\mathcal{I}) = F_P(\mathcal{E},\mathcal{I})$ for all $P \in \mathsf{P}^{\mathsf{N}}$ and so $F(\tilde{\mathcal{E}},\mathcal{I}) = F(\mathcal{E},\mathcal{I})$ by Eq. (1) in the main text . As $\tilde{\mathcal{E}}$ is covariant under Pauli channels, there exists a probability distribution $p(Q)$ over the set of Pauli matrices such that [3].

$$\tilde{\mathcal{E}}(A) = \sum_Q p(Q) Q A Q^\dagger. \tag{22}$$

For any Kraus operator decomposition, the process fidelity can be written as [5]

$$F(\tilde{\mathcal{E}},\mathcal{I}) = \sum_Q p(Q) |\operatorname{Tr} Q|^2/4^N = p(I). \tag{23}$$

Substituting Supplementary Equation (22) into Eq. (2) in the main text and using $[P,I]=0$, $p(Q)\geq 0$, and Supplementary Equation (23) gives

$$\begin{aligned} F_P(\tilde{\mathcal{E}},\mathcal{I}) &= \sum_{Q:[Q,P]=0} 2p(Q) - 1 \\ &\geq 2p(I) - 1 = 2F(\tilde{\mathcal{E}},\mathcal{I}) - 1. \end{aligned} \tag{24}$$

The lower bound follows as the $F_P(\tilde{\mathcal{E}},\mathcal{I})$ are eigenvalues of $\tilde{\mathcal{E}}$ and hence are in the unit disc [6]. □

Supplementary Note 5. Finite sampling effects

We now consider the effect of finite samples. Specifically, we will show that with appropriate choices of sequence lengths, the uncertainty in the estimate $\hat{F}$ obtained via Eq. (1) in the main text will scale as $O([1-F]/\sqrt{K})$ where the implicit constants are independent of the number of qubits. We will also show that if the experimental parameters are chosen appropriately, the implicit constant should be at most 1, that is, $\sigma \leq (1-F)/\sqrt{K}$. All the "approximately normal" statements in this section can be replaced by rigorous statements using the results of [7], Hoeffding's inequality [2] and the union bound, at the expense of additional notation and less favorable (but pessimistic) constants.

First, note that estimating the expectation value of the sequence labelled by l with a finite number of measurements R will produce an estimate of each expectation value $\langle f_{P,m,l}\rangle$ with an error $\epsilon_{P,m,l}$ that is approximately normally distributed with the standard deviation $\sigma_{P,m,l} \propto 1/\sqrt{R}$ independent of the number of qubits by the central limit theorem. Averaging the estimated expectation values $\langle f_{P,m,l}\rangle$ over a finite number L of random sequences will give an estimate of $\langle f_{P,m,l}\rangle$ with an error $\epsilon_{P,m}$ that is approximately normally distributed with standard deviation $\sigma_{P,m} \propto 1/\sqrt{L}$ that is independent of the number of qubits, again by the central limit theorem. Formally, the error in $\langle f_{P,m,l}\rangle$ can be divided into the average of a number of normally distributed random variables (the errors on the individual estimates), which contributes $O(1/\sqrt{LR})$ to $\sigma_{P,m}$, and the error from sampling a finite number of random sequences, which contributes $O(1/\sqrt{L})$ to $\sigma_{P,m}$. Hence, the error will be dominated by the finite number of random sequences. Using a series expansion of the ratio

$$\hat{F}_P := \left(\frac{\langle f_{P,m_2,l}\rangle + \epsilon_{P,m_2}}{\langle f_{P,m_1,l}\rangle + \epsilon_{P,m_1}}\right)^{1/\delta m},$$

the estimated process fidelity obtained by averaging $\hat{F}_P$ over K Pauli matrices will satisfy

$$\begin{aligned} \hat{F} &= \frac{1}{K}\sum_P \hat{F}_P \\ &\approx \sum_P \frac{F_P}{K} + \sum_P \frac{\delta\epsilon_P}{K\delta m}, \end{aligned} \tag{25}$$

where we define $\delta\epsilon_P = \epsilon_{P,m_2} - \epsilon_{P,m_1}$. Note that $\delta\epsilon_P$ is a difference between two approximately normal random variables with standard deviation $O(1/\sqrt{L})$ (neglecting the subleading term $O[1/\sqrt{LR}]$) and so $\mathbb{V}(\delta\epsilon_P) = O(1/L)$. Assuming that the $\delta\epsilon_P$ and F_P are independent, the expected variance of the estimate $\hat{F}$ over K Pauli matrices sampled uniformly with replacement is

$$\begin{aligned} \mathbb{V}^2(\hat{F}) &\approx K\,\mathbb{V}_P^2\left(\frac{F_P}{K}\right) + K\,\mathbb{V}^2\left(\frac{\delta\epsilon_P}{K\delta m}\right) \\ &\approx \frac{\mathbb{V}_P^2(F_P)}{K} + \frac{\mathbb{V}^2(\delta\epsilon_P)}{K\delta m^2}. \end{aligned} \tag{26}$$

We now show that both terms in Supplementary Equation (26) can be made to scale as $(1-F)^2/K$ by choosing parameters appropriately. The first term satisfies

$$\mathbb{V}^2(\hat{F}_P) \leq [1 - F(\mathcal{E},\mathcal{I})]^2 \tag{27}$$

since for any Pauli matrix P,

$$\begin{aligned} |F(\mathcal{E},\mathcal{I}) - F_P(\mathcal{E},\mathcal{I})| &\leq \max_{Q\in\mathsf{P}^{\mathsf{N}}} |F(\mathcal{E},\mathcal{I}) - F_P(\mathcal{E},\mathcal{I})| \\ &\leq 1 - F(\mathcal{E},\mathcal{I}) \end{aligned} \tag{28}$$

by Lemma 4. Furthermore, if the δm are chosen to be proportional to $1/(1-F)$, then the variance of $\hat{F}$ is proportional to $(1-F)^2$, so that we can efficiently estimate $1-F$ to multiplicative precision. The values of m in Supplementary Table 1 approximately satisfy this condition. With such choices of δm, we then have $\mathbb{V}^2(\hat{F}) = O[(1-F)^2/K]$. Furthermore, if L and R are sufficiently large so that $\mathbb{V}^2(\delta\epsilon_P)$ is negligible, than the variance of the estimator will satisfy

$$\mathbb{V}^2(\hat{F}) \leq (1-F)^2/K =: \sigma^2_{\text{Pauli}}. \tag{29}$$

It can be seen in Fig. (2) in the main text that the standard deviation decreases with the square-root of the

sampled subspaces K, with a least squares fit giving $\sigma = 0.0127(2)/\sqrt{K}$. This is consistent with the above analysis, which was based on the assumption that the $\delta\epsilon_P$ and F_P are independent. If we assume quantum projection noise ($\sqrt{p(1-p)/R}$) to be the only error source in the experiments, where p is the probability of measuring a certain outcome and R is the number of times a sequence is repeated, we can calculate a lower bound σ_{lower} for the measured data. This lower bound could be reached if the noise in the noise in the system is completely isotropic (e.g. global depolarizing). Biased noise or drift (see Supplementary Figure 3) will lead to uncertainties bigger than those originating from quantum projection noise.

The observed standard deviation σ is larger than the lower bound given by quantum projection noise $\sigma_{\text{lower}} = 0.00375(1)/\sqrt{K}$ but smaller than the upper bound $\sigma_{\text{Pauli}} = 0.0275(8)/\sqrt{K}$ on the contribution from sampling a finite number of Pauli matrices. This suggests that the other source of statistical uncertainty, namely, a finite number of randomizations L and measurements per sequence R, is sufficiently small to allow us to accurately estimate the process fidelity.

Supplementary Note 6. Correction operators for the MS gate

We performed cycle benchmarking for the identity and MS gates. The MS gate satisfies $\text{MS}^4 = \text{I}$, so that we can restrict m to be an integral multiple of 4. Indeed, $\text{MS}^2 \propto \text{X}^{\otimes\text{N}}$ so that we could restrict m to be even numbers by keeping track of the sign (which would depend on the Pauli matrix P). To compute the expectation value of $\mathcal{C}(P)$, we need to know how an arbitrary Pauli operator Q propagates through the MS gate. Using $\text{MS} \propto (I - iX^{\otimes N})/\sqrt{2}$ for even N gives

$$\mathcal{MS}(Q) = \text{MS}Q\text{MS}^\dagger = \begin{cases} Q & \text{if } QX^{\otimes N} = X^{\otimes N}Q \\ iQX^{\otimes N}\text{MS} & \text{otherwise.} \end{cases} \quad (30)$$

Supplementary Note 7. Experimental methods

The CB experiments are defined by a sequence of N-qubit Clifford gates according to the experimental protocol in Fig. (1) in the main text . We use two distinct types of Clifford gates, non-entangling and thus *local* Clifford gates (gates $\tilde{\mathcal{B}}$ in green and gates $\tilde{\mathcal{R}}$ in blue in Fig. (1) in the main text) and the fully entangling MS gates (gates $\tilde{\mathcal{G}}$ in red in Fig. (1) in the main text), which act on all N-qubits in the register simultaneously. A local Clifford gate consists of a set $\mathcal{R} = \bigotimes_{j=1}^{N} R(\theta)_j$ of individual single-qubit rotations $R(\theta)_j = \exp(-i\theta p_j/2)$, acting on qubit j with an angle θ, where $p_j \in [X, Y, Z]$ are single-qubit Pauli operations. The fully entangling MS gate is defined as $\text{MS} = \exp(-i\, S_x^2/8)$, with $S_x = X_1 + ... + X_N$.

After defining the sequences we compile them into the actual machine language [8]. In this experiment an elementary single qubit operation consist of one addressed z-rotation sandwiched between two collective rotations around the x- or y-axis, e.g. $X(\pi/2)_1 = X(-\pi/2)_{12}Z(\pi/2)_1X(\pi/2)_{12}$ for 2 qubits. The collective x- and y-rotations can be seen as simple basis changes on the entire register, and thus these basis changes can be shared by the individual qubit operations. By changing the temporal order of the collective x-, y-rotations and the individual z-rotations, the total number of collective rotations can be minimized.

We expect the single qubit z-rotations to have significantly larger infidelity compared to the collective rotations for the following reasons: First, the addressed laser beam has a smaller beam size and hence has larger intensity fluctuations. Second, we perform the z-rotations using the AC-Stark effect, which is quadratically more sensitive to intensity fluctuations than resonant x-, y-rotations. Therefore the number of single qubit rotations $Z(\theta)_j$ needed to perform a N-qubit Pauli operation is expected to be the limiting factor for local operations. In general, the average number of single qubit rotations per N-qubit Pauli operation scales linearly with N. To simplify the calibration procedure we only perform $Z(\pi/2)_j$ rotations. Thus e.g. a $Z(\pi)_j$ operation is implemented using two $Z(\pi/2)_j$ operations. In Supplementary Figure 1 we show the dependency of the average number of $Z(\pi/2)_j$ operations on the number of qubits. On average we implement $1.27(2) \cdot N$ addressed $\pi/2$ rotations for an N-qubit Pauli operation.

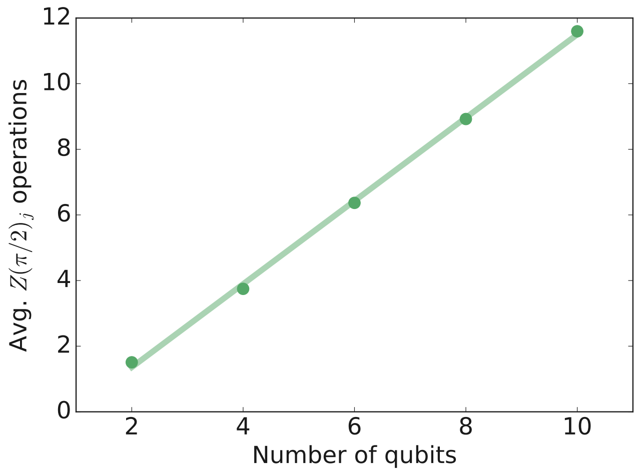

Supplementary Figure 1. Average number of $Z(\pi/2)_j$ operations needed to implement a N-qubit Pauli gate.

In Supplementary Table 1 we give an overview of the experimental parameters that we used to estimate the local CB and the dressed MS fidelities. The number of sub-

Supplementary Table 1. Experimental parameters for the taken CB data for different register. Since we implement each sequence twice, once with and once without interleaved MS gates, the total number of sequences is $2 \cdot K \cdot 2(\text{or } 3) \cdot L$, where each individual sequence is repeated R times.

Qubits	Subspaces K	Sequence lengths m	Random sequences L	Total sequences	Repetitions R	Measurement time (h)
2	15	4, 40	10	600	100	2.6
4	255	4, 20	10	10200	100	15.7
6	43	4, 8, 12	10	2580	100	3.4
8	24	4, 8	10	960	100	2.0
10	21	4, 8	10	840	100	1.9

spaces K describes the number of individual Pauli channels we explore. For registers containing 2 and 4 qubits we measure all possible $4^N - 1$ subspaces, excluding the identity. As the success probability decays exponentially with the sequence length m, it is sufficient to measure two sequences lengths (see Supplementary Note 4). In the case of 6-qubits we measure 3 different sequence lengths to perform model test analysis (see Supplementary Note 8). For each sequence length m we measure L different random sequences. We implement each sequence twice, once with and once without interleaved MS gates, hence the total number of sequences is $2 \cdot K \cdot 2(\text{or } 3) \cdot L$. Since each sequence implementation ends with a projective measurement into one single quantum state, we repeat every sequence $R = 100$ times to measure the outcome probabilities.

Supplementary Note 8. Testing the dependence of the estimator on the sequence length

If the noise in the system is Markovian, we expect the estimated process fidelity to be independent of the sequence lengths m_1 and m_2 to within $\mathcal{O}([1 - F_{\text{RC}}(\tilde{\mathcal{G}}, \mathcal{G})]^2)$ (see Theorem 3). We test this by performing measurements at 3 different sequence lengths for 6 qubits, as described in Supplementary Table 1. We validate that the estimated process fidelity is independent of m_1 and m_2 by comparing the results of three different length pairs 4-8, 4-12 and 8-12. As can be seen in Supplementary Table 2, the measured fidelities agree to within half a standard deviation, which supports the validity of the assumtions for our experimental apparatus.

Supplementary Table 2. 6-qubit process fidelities estimated via CB (%) using different pairs sequence lengths (m_1, m_2). The results illustrate that the estimated process fidelity is independent of the sequence lengths used, subject to the constraint in step 2 of the protocol.

(m_1, m_2)	Local gates	Dressed MS gate
(4,8)	97.0(2)	91.3(5)
(4,12)	97.0(2)	91.2(4)
(8,12)	96.9(4)	91.3(8)

Supplementary Note 9. Testing the dependence of the fidelity uncertainty on the register size

As we have shown in Supplementary Note 5, the variance of the fidelity estimate is independent of the number of qubits N. We experimentally test this prediction by analyzing the variance of the measured fidelities as a function of the register size. Since the process fidelity is reduced for larger register sizes, the variances themselves are expected to differ. We therefore investigate the ratio between the observed variance and the bound of equation Supplementary Equation (29). In Supplementary Figure 2 we plot the ratio between the standard deviation σ of the measured fidelity and the upper bound σ_{Pauli} from sampling a finite number of Pauli channels (see Supplementary Note 5) against the number of qubits N in the register. In the data we cannot observe a clear trend or dependency of the observed variance relative to the worst-case bound, supporting the claim that the uncertainty of the fidelity estimate is independent of the register size.

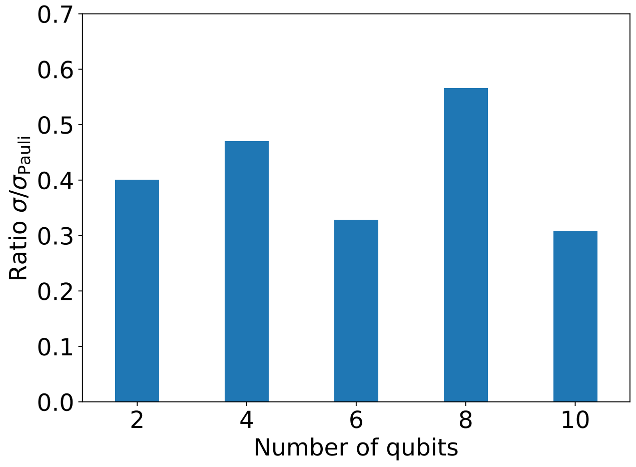

Supplementary Figure 2. Ratio between the uncertainty on the fidelity estimate σ and the theoretical bound from sampling Pauli channels σ_{Pauli} against the register size.

Supplementary Note 10. Analyzing fidelity drift

Slow temperature fluctuations on the timescale of minutes to days cause changes in various components of our experimental apparatus. One of the major causes for a loss in fidelity over time is the alignment of the laser beams relative to the ion position. The single ion addressing laser beam is tightly focused to a spot size of $\sim 2\,\mu$m and the beam position changes as the temperature varies. This change in position leads to a miscalibration of the Rabi frequency as well as an increase in intensity fluctuations. We analyze the temporal dependence of the fidelity with 4-qubit CB as depicted in Supplementary Figure 3. The 255 subspaces were measured in 3 sessions, where the experimental system was recalibrated at the beginning of each session. We approximate the drift of the fidelity to be linear in first order and thus can describe the time dependent fidelity as $F(t) = F_0 - \epsilon t$. We obtain an average loss of fidelity of $\epsilon_\mathrm{L} = 3.3(5) \cdot 10^{-3}\,\mathrm{h}^{-1}$ for local gates and $\epsilon_\mathrm{I} = 5.4(8) \cdot 10^{-3}\,\mathrm{h}^{-1}$ for the dressed MS gate, see Supplementary Table 3. This measurement suggests that we can expect a maximum loss of fidelity of 1 % when recalibrating the apparatus every two hours.

Supplementary Table 3. 4-qubit fidelity drift rates, where ϵ_L and ϵ_D describe the loss of fidelity per hour for local gates and the dressed MS gate. The data corresponds to the estimated linear slopes of Supplementary Figure 3

Session	ϵ_L (h^{-1})	ϵ_D (h^{-1})
1	$3.9(8) \cdot 10^{-3}$	$8.9(1.5) \cdot 10^{-3}$
2	$3.1(5) \cdot 10^{-3}$	$3.2(6) \cdot 10^{-3}$
3	$3.0(1.1) \cdot 10^{-3}$	$4.2(1.7) \cdot 10^{-3}$
Average	$3.3(5) \cdot 10^{-3}$	$5.4(8) \cdot 10^{-3}$

SUPPLEMENTARY REFERENCES

[1] Joel J. Wallman and Joseph Emerson, "Noise tailoring for scalable quantum computation via randomized compiling," Phys. Rev. A **94**, 052325 (2016).

[2] Wassily Hoeffding, "Probability Inequalities for Sums of Bounded Random Variables," Journal of the American Statistical Association **58**, 13 (1963).

[3] A. S. Holevo, "Additivity Conjecture and Covariant Channels," International Journal of Quantum Information **03**, 41 (2005).

[4] Jonas Helsen, Xiao Xue, M.K. Vandersypen, Lieven, and Stephanie Wehner, "A new class of efficient randomized benchmarking protocols," arXiv:1806.02048 (2018).

[5] Michael A. Nielsen, "A simple formula for the average gate fidelity of a quantum dynamical operation," Physics Letters A **303**, 249 (2002).

[6] David E Evans and R. Hoegh-Krohn, "Spectral Properties of Positive Maps on C^*-Algebras," Journal of the London Mathematical Society **17**, 345–355 (1978).

[7] Robin Harper, Ian Hincks, Chris Ferrie, Steven T. Flammia, and Joel J. Wallman, "Statistical analysis of randomized benchmarking," arXiv:1901.00535 (2019).

[8] Esteban A Martinez, Thomas Monz, Daniel Nigg, Philipp Schindler, and Rainer Blatt, "Compiling quantum algorithms for architectures with multi-qubit gates," New J. Phys. **18**, 063029 (2016).

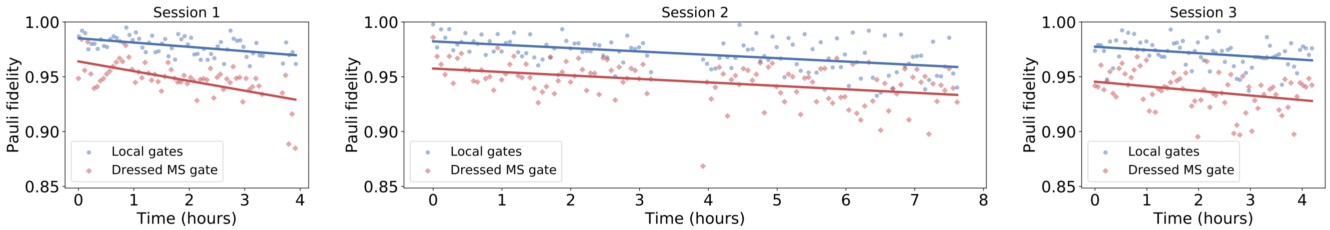

Supplementary Figure 3. 4-qubit Pauli fidelities for local gates (blue) and the dressed MS gate (red) plotted on the time in hours. We measured all 255 subspaces in three measurement sessions, where the experiment was recalibrated at the beginning of each session.
